# Injectable biomaterial induces regeneration of the intervertebral disc in a caprine loaded disc culture model†

**DOI:** 10.1039/d3bm00150d

**Published:** 2023-05-17

**Authors:** Joseph W. Snuggs, Kaj S. Emanuel, Christine Rustenburg, Ronak Janani, Simon Partridge, Christopher Sammon, Theo H. Smit, Christine L. Le Maitre

**Affiliations:** a Department of Oncology and Metabolism, Medical School, The University of Sheffield Sheffield UK c.lemaitre@sheffield.ac.uk; b Biomolecular Sciences Research Centre, Sheffield Hallam University Sheffield UK; c Amsterdam UMC, University of Amsterdam, Department of Orthopedic Surgery and Sports Medicine, Amsterdam Movement Sciences Amsterdam the Netherlands; d Department of Orthopedic Surgery, CAPHRI Care and Public Health Research Institute, Maastricht University Medical Center+ Maastricht the Netherlands; e Materials Engineering Research Institute, Sheffield Hallam University Sheffield UK

## Abstract

Back pain is the leading cause of disability with half of cases attributed to intervertebral disc (IVD) degeneration, yet currently no therapies target this cause. We previously reported an *ex vivo* caprine loaded disc culture system (LDCS) that accurately represents the cellular phenotype and biomechanical environment of human IVD degeneration. Here, the efficacy of an injectable hydrogel system (LAPONITE® crosslinked pNIPAM-*co*-DMAc, (NPgel)) to halt or reverse the catabolic processes of IVD degeneration was investigated within the LDCS. Following enzymatic induction of degeneration using 1 mg mL^−1^ collagenase and 2 U mL^−1^ chondroitinase ABC within the LDCS for 7 days, IVDs were injected with NPgel alone or with encapsulated human bone marrow progenitor cells (BMPCs). Un-injected caprine discs served as degenerate controls. IVDs were cultured for a further 21 days within the LDCS. Tissues were then processed for histology and immunohistochemistry. No extrusion of NPgel was observed during culture. A significant decrease in histological grade of degeneration was seen in both IVDs injected with NPgel alone and NPgel seeded with BMPCs, compared to un-injected controls. Fissures within degenerate tissue were filled by NPgel and there was evidence of native cell migration into injected NPgel. The expression of healthy NP matrix markers (collagen type II and aggrecan) was increased, whereas the expression of catabolic proteins (MMP3, ADAMTS4, IL-1β and IL-8) was decreased in NPgel (±BMPCs) injected discs, compared to degenerate controls. This demonstrates that NPgel promotes new matrix production at the same time as halting the degenerative cascade within a physiologically relevant testing platform. This highlights the potential of NPgel as a future therapy for IVD degeneration.

## Abstract 2

Here, the efficacy of an injectable hydrogel system (NPgel), either alone or together with progenitor cells, was investigated within a degenerate loaded disc model. NPgel inhibited cellular catabolism and promoted anabolism, supporting regeneration.

## Graphical abstract

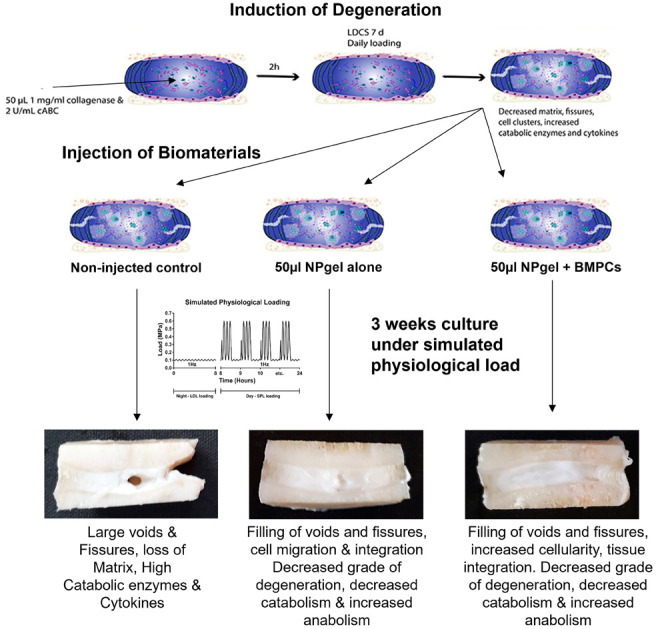

## Introduction

Low back pain (LBP) remains one of the main causes of long-term disability and brings about a high socio-economic burden worldwide.^1,2^ Whilst the causes of chronic LBP are multifactorial, approximately half of all cases are attributed to degeneration of the intervertebral disc (IVD).^3–5^ The physiology of the IVD is finely balanced; tissue catabolism is matched by anabolism in order to maintain mechanical integrity which enables the functions of the IVD, allowing movement and withstanding the compressive forces of the spine.^6–8^ Yet, during degeneration NP cell phenotype shifts towards catabolism, which is characterised by multiple factors such as increased expression of cytokines,^9–13^ altered matrix composition and degradation,^14–19^ increased apoptosis and senescence,^20–23^ and nerve and blood vessel ingrowth.^24–29^ Therefore, the overall balance is driven towards tissue destruction, leading to the eventual loss of function and the onset of LBP.^30–32^ However, to date the majority of treatments focus on pain management, physical therapy, and surgical removal of herniated tissue, with none targeting the underlying pathophysiological causes of IVD degeneration.^33,34^ Targeting the causes of IVD degeneration would be beneficial for future therapies, as halting the catabolic destruction of tissue, regenerating the structural integrity, and restoration of normal mechanics of the IVD may alleviate the individual and societal burden of LBP.

Potential new therapies that employ a biological and/or mechanical approach to IVD degeneration are now emerging, which would have the clinical aims of restoring disc integrity and correcting spine biomechanics.^33–35^ Tissue engineering strategies such as the use of biomaterial scaffolds,^36–40^ the implantation of regenerative cell sources,^41–44^ or a combination of both in unison,^45–48^ are attractive as they may have the ability to target a number of underlying biological causes, promoting matrix regeneration whilst also restoring disc height and biomechanics. As biomechanics and cell biology are strongly interdependent,^32^ successful regenerative therapies should not only intervene at a cellular level but target the biomechanical environment of the disc cells as well.

We have previously reported the development and characterisation of NPgel; a synthetic, injectable, LAPONITE® crosslinked poly-*N*-isopropylacrylamide-*co-N*,*N*′-dimethylacrylamide (pNIPAM-*co*-DMAc) hydrogel and its potential as a tissue engineering approach to treat IVD degeneration.^46,47,49,50^ Fully synthesised NPgel exists as a free-flowing liquid suspension which irreversibly solidifies at a specific, pre-engineered temperature (37 °C) into a 3-dimensional hydrated polymer.^50^ NPgel has the ability to differentiate incorporated human bone marrow progenitor cells (BMPCs) into nucleus pulposus (NP)-like cells *in vitro*, which express phenotypic NP markers and promote matrix deposition that mimic native NP tissue, without the need of any additional growth factors.^46,47,49^ We have also demonstrated the injectability of NPgel through small-bore needles (26G) into bovine NP tissue explants, which provides the advantage of being able to accurately administer NPgel into the disc without causing further damage to the tissue.^47^ In addition, BMPCs encapsulated within NPgel were still able to differentiate into NP-like cells when injected into bovine NP explants and biomechanical function was restored due to the integration of NPgel into the tissue.^47^ Further studies have displayed that NPgel affords protection to BMPCs when cultured within a degenerate environment and is still able to facilitate their differentiation into NP-like cells.^51^ Taken together, previous studies highlight that NPgel has the ability to integrate into IVD tissue and restore biomechanics, enable the introduction of a healthy, regenerative cell source and may confer protection within the degenerate environment they are introduced to,^46,47,49^ indicating the promising potential of NPgel as a therapy.

However, to determine the efficacy of NPgel to evoke regenerative effects under physiological conditions, further testing of NPgel, particularly within more complex model systems, must be undertaken to validate the efficacy of NPgel within *in vivo*-like environments. Multiple animal models have been developed in such testing to obtain an understanding of IVD biology, degeneration and to evaluate the safety and efficacy of potential interventions.^52–54^ The use of animal models, however, have practical, ethical and biological limitations. Therefore, loaded disc culture systems (LDCS) have been developed,^55^ whereby intact IVDs or motion segments are excised from animals obtained from the abattoir and cultured within bioreactors. Tissues obtained and cultured in this way enable *ex vivo* experiments to be performed under highly reproducible and controlled environments, and allow accurate mimicry of the human IVD, such as diurnal biomechanical loading patterns and induction of degeneration, which are difficult to replicate within quadruped animal models.^8,56^ To this end, we previously established an *ex vivo* caprine LDCS to accurately model the catabolic environment of the degenerate human IVD over a 7-day period, where degeneration was induced by enzymatic degradation using 1 mg mL^−1^ collagenase and 2 U mL^−1^ chondroitinase ABC.^56^ This model successfully replicated many of the factors associated with human IVD degeneration, such as increased cytokine, degradative enzyme and catabolic protein expression together with decreased matrix deposition and protein expression,^56^ and has the potential to allow the rapid, reproducible testing of new innovations without relying on the use of animal models prior to animal and human trials.

Therefore, this study aimed to further test the efficacy of NPgel (±BMPCs) in modulating the catabolic changes that occur during IVD degeneration within the *ex vivo* caprine LDCS. Specifically, this study tested the hypothesis that NPgel injection into caprine IVDs, following the onset of degeneration within the LDCS, would integrate with native tissue, halt the over-expression of catabolic proteins associated with degeneration, increase the production and deposition of healthy NP matrix markers and decrease the overall grade of IVD degeneration, hereby initiating the regenerative process. We investigated the injection of NPgel alone or NPgel containing BMPCs in this study to assess the importance of simultaneously administering a new stem cell source alongside a scaffold, which may facilitate tissue integration and provide a healthy cell source able to proliferate, differentiate and regenerate the disc. Together, the investigations here aimed to determine whether NPgel has the ability to halt degenerative processes within an *ex vivo* model system mimicking human IVD degeneration and be used as both an integrated tissue scaffold and a cell delivery vehicle.

## Methods

### Caprine IVD isolation

Lumbar spines (T12-L5) from skeletally mature female Dutch milk goats (3–5 years old, *n* = 4) were obtained from a local abattoir (Amsterdam, NL) and sterilised with medical grade iodide–alcohol solution. Within 24 hours of slaughter, excessive soft and connective tissue was removed and individual IVDs with adjacent cartilaginous endplates were dissected in two parallel transverse planes using an oscillating surgical saw. Dissected motion segments (*n* = 17) were cleaned with sterile gauze and rinsed in phosphate buffered saline solution (PBS) and ethanol.

### NPgel formulation

NPgel was prepared as previously described.^46^ Briefly, 10 ml exfoliated suspension of 0.11 g LAPONITE® clay nanoparticles (25–30 nm diameter, <1 nm thickness) (BYK Additives Ltd, Cheshire, UK) was prepared in ultra-pure water. 0.87 g *N*-isopropylacrylamide 99% (NIPAM) (KJ Chemicals Corporation, Japan), 0.13 g *N*,*N*′-dimethylacrylamide (DMAc) (Sigma, Gillingham, UK) and 0.01 g 2,2′-azobisisobutyronitrile (AIBN) (Sigma, Gillingham, UK) were added to the LAPONITE® suspension, mixed well and strained through 5–8 μm pore filter paper. The mixture was polymerised at 80 °C for 24 h and subsequently cooled to 38–40 °C before the addition of cells.

### Bone marrow progenitor cell isolation and culture

Human femoral heads were collected from Royal Hallamshire Hospital, Sheffield, UK, following hip replacement surgery for the treatment of osteoarthritis (samples sourced with ethical approval from South Yorkshire and North Derbyshire Musculoskeletal Biobank (REC approval 15/SC/0132, HTA licence 12182) (*n* = 3). Bone marrow was extracted by tissue biopsy punch and washing in cell culture media. Bone marrow aspirate (in cell culture media) was filtered using a 100 μm cell strainer before being carefully layered onto an equal amount of Histopaque®-1077 (Sigma) (*e.g.*, 3 mL bone marrow aspirate was layered onto 3 mL Histopaque®-1077) and centrifuged at 400*g* for 30 min at room temperature. Using a Pasteur pipette, the opaque layer at the Histopaque-media interface (containing mononuclear cells) was taken. The cells were washed in 10 mL media and centrifuged at 400*g* for 5 min. Washing and centrifugation was repeated two additional times. Bone marrow progenitor cells (BMPC) were isolated by removing CD45^+^ cells (haematopoietic cells) from the solution by using CD45 MicroBeads (130-045-801, Miltenyi Biotec, Bergisch Gladbach, Germany) with magnetic activated cell sorting (MACS) using a MiniMACS™ starting kit (130-090-312, Miltenyi Biotec) following the manufacturer's protocol. The unlabelled cell fraction (CD45^−^), containing BMPCs, was collected and centrifuged at 400*g* for 5 min. The cell pellet was finally resuspended in cell culture media and cultured in T75 tissue culture flasks (Nunc) at 37 °C with 5% (v/v) CO_2_ in a humidified environment.

### Loaded disc culture system – induction of IVD degeneration and NPgel ±BMPC injection

The loaded disc culture system (LDCS) consists of individual culture chambers, enabling IVD culture under dynamic loading conditions, that are placed in an incubator at 37 °C. Firstly, caprine IVDs were pre-loaded for 3 days before injection of a combination of 50 μL 1 mg mL^−1^ collagenase (Invitrogen, Paisley, UK) and 2 U mL^−1^ chondroitinase ABC from *Proteus vulgaris* (Sigma) using a 26G needle and subjected to simulated physiological loading (SPL) for 7 days to establish the degenerate model (ESI Fig. 1†).^56^ SPL consisted of 8 hours of night-time loading of 50 ± 10 N (0.09–0.11 MPa) 1 Hz, and 16 hours of alternating half hours of 150 ± 100 N (0.1–0.6 MPa) 1 Hz and 50 ± 10 N (0.09–0.11 MPa) 1 Hz (ESI Fig. 1†), mimicking *in vivo* compressive forces during walking and sleeping of the species as reported previously.^57,58^ SPL was previously validated^8^ to maintain cell viability, gene expression and biomechanical properties of caprine IVDs. Following 7 days of degeneration induction, IVDs were injected (∼50 μL injection volume, using 26G needles) with NPgel alone (*n* = 5) or containing BMPCs at a density of 4 × 10^6^ cells per mL (NPgel + BMPC, *n* = 4), un-injected IVDs served as degeneration controls (*n* = 6). Subsequently, IVDs were cultured under SPL for a further 21 days prior to assessment. IVDs were cultured in standard DMEM (Gibco, Paisley, UK) with 10% FBS (HyClone, Logan, UT, USA), 4.5 g L^−1^ glucose (Merck, Darmstadt, Germany), 25 mmol L^−1^ HEPES buffer (Invitrogen), 50 μg mL^−1^ ascorbate-2-phosphate (Sigma Aldrich, St Louis, MO, USA), 10 000 U mL^−1^ penicillin, 10 mg mL^−1^ streptomycin and 25 μg mL^−1^ amphotericin B (Gibco, Paisley, UK). Each culture chamber contained 50 mL of culture medium which circulated continuously with 3 mL h^−1^ using a peristaltic pump and was changed every 3 or 4 days, after flushing with 25 mL of PBS per culture chamber. During the whole culture period, the IVDs were loaded with dynamic axial loading consisting of SPL.^8^

### Caprine IVD harvesting and histological grading

Following completion of the loading regime in the LDCS, IVDs were removed from their culture chamber and fixed in 10% neutral buffered formalin (Leica Biosystems, Nussloch, Germany) for 1 week and thereafter decalcified using decalcifier II (Leica Biosystems, Nussloch, Germany), also for 1 week. Tissue slices of ∼3 mm thick were cut paramidsagittally from the IVD using a scalpel and embedded in paraffin wax. Sections (4 μm) were cut and mounted onto positively charged slides (Leica Biosystems, Newcastle, UK). The extent of histological disc degeneration was determined using histological grading as recommended for large animal IVD models.^59^ Namely, key features of IVD degeneration including cellular clusters, NP cell loss and necrosis, matrix staining, AF morphology, AF cellularity and ECM metaplasia, presence of tears and cleft formation and CEP morphology, *via* haematoxylin & eosin (H&E), Masson trichome and alcian blue staining, were investigated.^59^ As BEPs were removed prior to analysis, grading did not include bone remodelling scores.

### Histology to determine NPgel integration and cell migration

H&E (tissue morphology), Masson trichrome (collagen deposition) and alcian blue (presence of glycosaminoglycans, GAGs) staining was utilised to identify the structure of IVD tissues, location, integration and native NP cell migration into injected NPgel following previously published methods.^16,46,47,60^

### Immunohistochemistry to determine protein expression

Immunohistochemistry (IHC) was used to determine the cellular changes induced by enzyme degradation in the degenerated goat discs compared to degenerated discs with NPgel ±BMPCs injected into the NP region following the initial onset of degeneration within the LDCS. Extracellular matrix proteins, matrix degrading enzymes, key cytokines and factors which lead to nerve and blood vessel ingrowth were investigated using IHC following previously published methods^61^ using antibodies stated in Table 1. All cells within the field of view were counted as either immunopositive (brown staining) or immunonegative (only purple nuclei staining) until a total of 200 cells per sample had been counted. Counting was performed on cells present throughout the entirety of the NP tissue region, to determine the number of cells expressing each protein across the whole NP region. The percentage of immunopositive NP cells (for each target) was then determined.

**Table 1:** List of antibodies and methods used for immunohistochemistry (IHC) experiments in this study. All secondary antibodies used at a dilution of 1 : 500.^58^ All antibodies purchased from Abcam

Target	Clonality	Dilution	Antigen retrieval method	Secondary antibody
Collagen type II (ab34712)	Rabbit polyclonal	1 : 200	Enzyme	Goat anti rabbit (ab6720)
Aggrecan (ab3778)	Mouse monoclonal	1 : 100	Heat	Rabbit anti mouse (ab6727)
MMP3 (ab53015)	Rabbit polyclonal	1 : 400	Enzyme	Goat anti rabbit (ab6720)
ADAMTS4 (ab185722)	Rabbit polyclonal	1 : 200	None	Goat anti rabbit (ab6720)
IL-1β (ab9722)	Rabbit polyclonal	1 : 100	Heat	Goat anti rabbit (ab6720)
IL-8 (ab7747)	Rabbit polyclonal	1 : 100	Heat	Goat anti rabbit (ab6720)
VEGFA (ab52917)	Rabbit monoclonal	1 : 100	Enzyme	Goat anti rabbit (ab6720)
NGF (ab52918)	Rabbit monoclonal	1 : 100	Enzyme	Goat anti rabbit (ab6720)

### Statistics

Histological grade of degeneration and IHC immunopositivity data were not normally distributed, as values were within fixed scales, thus Kruskall–Wallis with Dunn's multiple comparisons tests were performed (Prism v8.1.1) to identify significant differences in cellular immunopositivity across treatment groups. A *p* value of <0.05 was considered statistically significant.

## Results

### Macroscopic appearance and histological grading of caprine IVDs

**Fig. 1:**
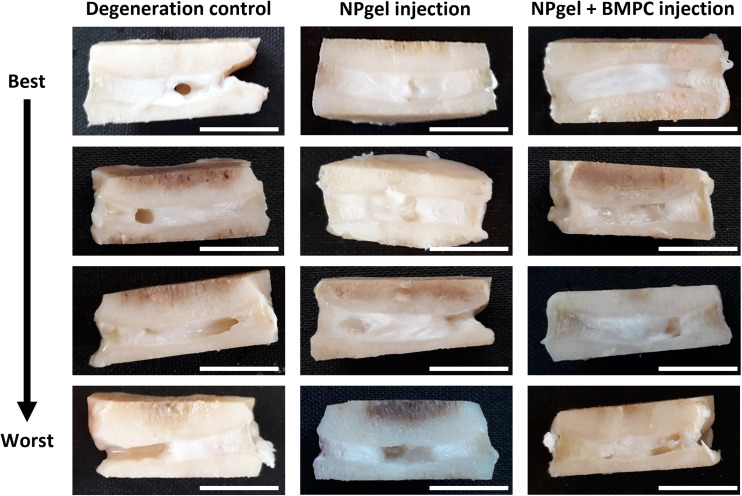
Macroscopic images of caprine intervertebral discs (IVD) following 31 days within the loaded disc culture system (LDCS). Representative best-to-worst case macroscopic images of caprine IVDs after NPgel injection (± bone marrow progenitor cells, BMPCs) and 21 days LDCS culture (following 3 days pre-load, 7 days enzymatic degeneration). NPgel appears ‘shiny’ and has integrated within nucleus pulposus tissue, where fissures are present, upon gelation within the central area of the disc.

**Fig. 2:**
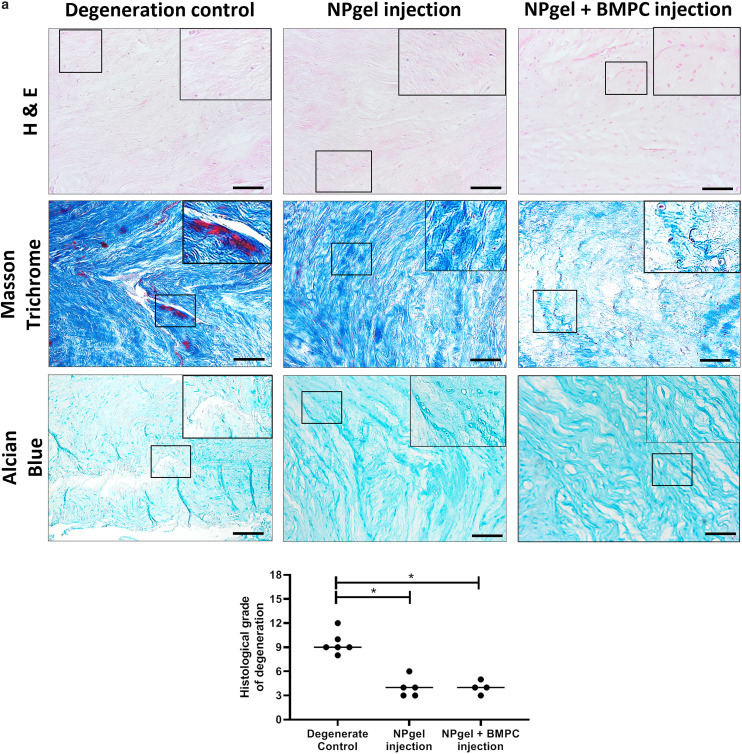
Histological staining and grading of caprine intervertebral discs (IVD) following 31 d within the loaded disc culture system (LDCS). Representative histological images of caprine IVDs after NPgel injection (± bone marrow progenitor cells, BMPCs) and 21 days LDCS culture (following 3 days pre-load, 7 days enzymatic degeneration). Utilising haematoxylin and eosin (H & E) (purple: nuclei, pink: cytoplasm and general tissue structure), Masson trichrome (blue/black: nuclei, blue: collagen, red: cytoplasm and other matrix/tissue, *e.g.*, muscle) and alcian blue (red: nuclei, blue: glycosaminoglycans) staining allows the identification of degenerative features within the IVDs. (a) Histological grading determined that the average grade of degeneration of NPgel (grade 4 = non-degenerate) and NPgel + BMPC (grade 4 = non-degenerate) injected discs was significantly decreased compared to degenerate controls (grade 9.5 = moderately degenerate) *p* ≤ 0.05. Horizontal lines represent median values. Scale bar 200 μm.

Following culture, fissures and destruction of NP tissue were observed macroscopically within degeneration control IVDs (Fig. 1). NPgel, whether injected either alone or with BMPCs incorporated, infiltrated and filled fissures within the NP region of IVDs, and was not extruded from IVDs during loading (Fig. 1). Histological grade of IVD degeneration, scored using a standardised system,^62^ was significantly decreased in both NPgel-injected (grade 3–6, median 4) (*p* = 0.0115) and NPgel + BMPC-injected (grade 3–5, median 4) (*p* = 0.0321) caprine IVDs compared to degenerate controls (grade 8–12, median 9.5) (Fig. 2a).

### NPgel integration, cell migration and increased cellularity following NPgel injection

**Fig. 3:**
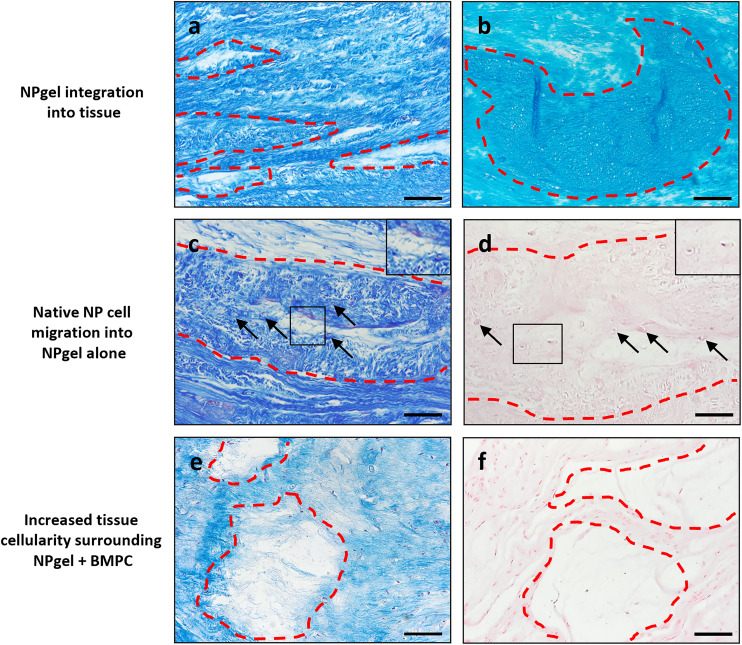
NPgel integration, native cell migration and increased cellularity following NPgel injection, alone or with incorporated bone marrow progenitor cells (±BMPC), and 31 days culture within the LDCS. Representative histological staining images identifying NPgel present within the NP region of caprine IVDs (red dashed areas), (a, c and e) Masson trichrome, (b) alcian blue, (d and f) haematoxylin & eosin staining. Staining (a, NPgel alone and b, NPgel + BMPC) highlights that following injection, NPgel is retained, fills void spaces and fissures within NP regions and integrates with IVD tissue. (c and d) Where NPgel, without BMPCs, has been injected, the infiltration of native NP cells into NPgel is observed (arrows and inset images, cell nuclei stained (c) red or (d) purple). (e and f) Following NPgel (+BMPCs) injection, the NP region surrounding the gel shows evidence of increased cellularity, indicating the successful delivery of a potentially regenerative cell source into the degenerate IVD. Scale bar a–c, e and f 100 μm, d 50 μm.

Collagen and GAG deposition was observed by Masson trichrome and alcian blue staining, respectively, within NP tissue where NPgel (±BMPCs) had been injected (red, dashed boxes) (Fig. 2 and 3a, b). NPgel (±BMPCs) also filled fissures and void spaces within the tissue, which are still present in degeneration controls (Fig. 2 and 3a, b). Where NPgel has been injected (without BMPCs) there was evidence of native NP cell migration into the hydrogel, further aiding the integration process (Fig. 3c and d, native cells within NPgel, red dashed boxes, are indicated by black arrows and inset images). Cells were also visualised within the NPgel region of NPgel + BMPC injected discs however the origin of these cells could not be specified. Also, following NPgel injection (+BMPCs), the regions of NP tissue adjacent to the gel showed increased cellularity, potentially indicating the successful delivery and migration of the new regenerative cell source into the disc (Fig. 3e and f, red and purple cell nuclei respectively). However, no cell tracking dyes were added to the BMPCs in this study, so the origin of these cells is impossible to determine. Increased cellularity, therefore, may also be due to a trophic effect of the NPgel or BMPCs within, to attract or stimulate the resident NP cells surrounding the biomaterial.

### Extracellular matrix protein expression

**Fig. 4:**
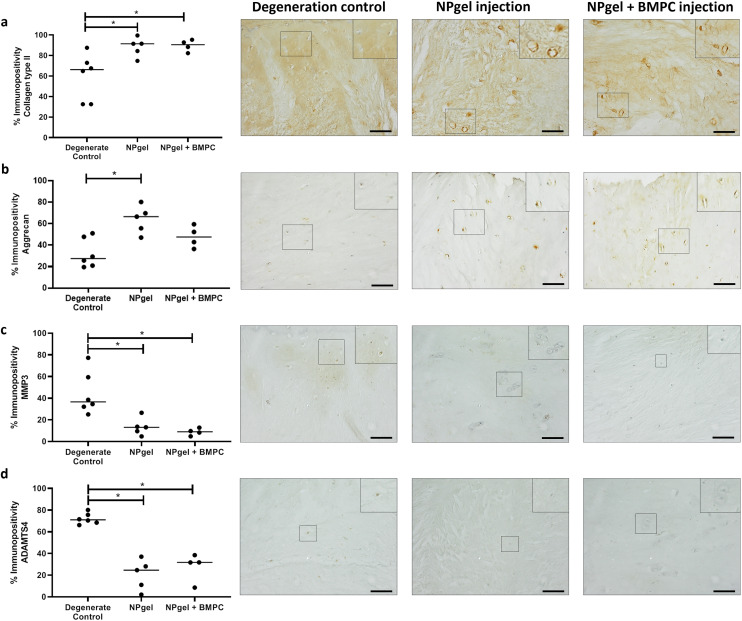
Extracellular matrix and degradative enzyme expression within caprine NP tissue. Expression of (a) collagen type II, (b) aggrecan, (c) MMP3 and (d) ADAMTS4 within native NP cells in caprine IVDs. Expression of proteins was determined by immunohistochemistry (IHC) after 21 d within the loaded disc culture system (LDCS) (following 3 d pre-load, 7 d enzymatic degeneration). Significant differences in percentage expression across degeneration control, NPgel injection and NPgel + BMPC injection was determined by counting the number of positive cells (brown stain) and negative cells (blue, nuclear haematoxylin stain only) to a total of 200 cells per sample, *p* ≤ 0.05. IHC images with inlets show representative staining patterns within NP tissue across the three groups for each individual protein of interest. Horizontal lines represent median values. Scale bar 200 μM.

The percentage of cells expressing collagen type II in NPgel-injected (median 91.4%, *p* = 0.0362) and NPgel + BMPC-injected (median 90.6%, *p* = 0.0306) caprine IVDs was also significantly increased compared to degenerate controls (median 66.1%) (Fig. 4a). The percentage of NP cells positive for aggrecan expression in NPgel-injected (median 66.5%) caprine IVDs was significantly increased compared to degenerate controls (median 27.5) (*p* = 0.0112) (Fig. 4b). An increase was also seen in NPgel + BMPCs (median 47.6%), but this failed to reach significance (*p* = 0.3879) (Fig. 4b).

### Cytokine and catabolic protein expression

**Fig. 5:**
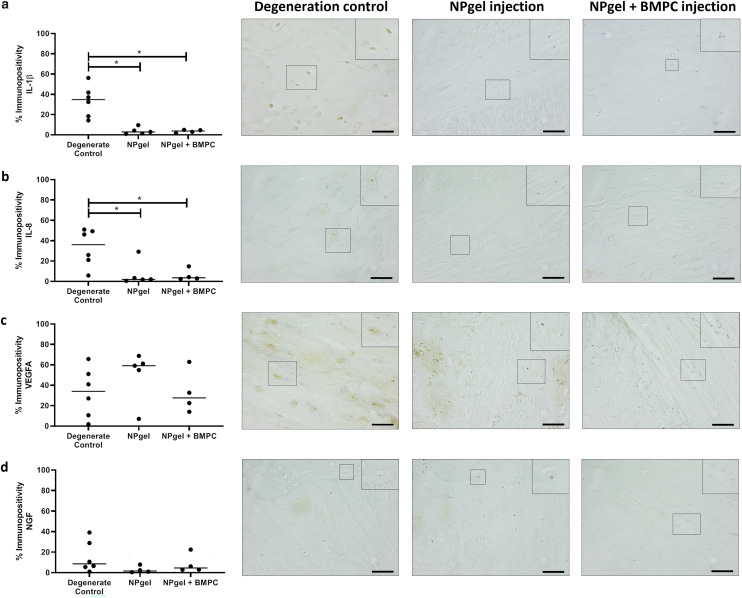
Cytokine, angiogenic and neurotrophic protein expression within caprine NP tissue. Expression of (a) IL-1β, (b) IL-8, (c) VEGFA and (d) NGF within native NP cells in caprine IVDs. Expression of proteins was determined by immunohistochemistry (IHC) after 21 d within the loaded disc culture system (LDCS) (following 3 d pre-load, 7 d enzymatic degeneration). Significant differences in percentage expression across degeneration control, NPgel injection and NPgel + BMPC injection was determined by counting the number of positive cells (brown stain) and negative cells (blue, nuclear haematoxylin stain only) to a total of 200 cells per sample, *p* ≤ 0.05. IHC images with inlets show representative staining patterns within NP tissue across the three groups for each individual protein of interest. Horizontal lines represent median values. Scale bar 200 μM.

The number of MMP3 immunopositive NP cells was significantly reduced in NPgel-injected (median 13.1%, *p* = 0.0387) and NPgel + BMPC-injected (median 9%, *p* = 0.0078) caprine IVDs when compared to degenerate controls (median 36.5%) (Fig. 4c). The number of NP cells immunopositive for ADAMTS4 was significantly decreased in NPgel-injected (median 24.7%, *p* = 0.0044) and NPgel + BMPC-injected (median 31.8%, *p* = 0.0487) caprine IVDs when compared to degenerate controls (median 71%) (Fig. 4d). The number of NP cells expressing IL-1β in both NPgel-injected (median 2.9%, *p* = 0.0056) and NPgel + BMPC-injected (median 3.8%, *p* = 0.0387) caprine IVDs was significantly decreased compared to degenerate controls (median 34.8%) (Fig. 5a). The number of NP cells expressing IL-8 in NPgel-injected (median 2%, *p* = 0.0188) and NPgel + BMPC-injected (median 3.6%, *p* = 0.0127) caprine IVDs was significantly decreased when compared to degenerate controls (median 36%) (Fig. 5b). There were no significant differences in the number of NP cells expressing either VEGF (Fig. 5c) or NGF (Fig. 5d) across all treatment groups.

## Discussion

This study aimed to investigate the injection of NPgel into degenerated caprine IVDs and determine its ability to integrate into the tissue, modulate the expression of healthy matrix markers and catabolic proteins, and decrease the overall grade of degeneration, all within the confines of our caprine LDCS that mimics the cellular phenotype observed during human degenerative processes.^56^ The data presented here indicate that following injection and 21 days of culture, after the initial 7-day process to initiate degeneration, NPgel (±BMPCs) fully integrated with caprine IVD tissue and increased the expression of healthy NP matrix (collagen type II, aggrecan), decreased expression of catabolic proteins including cytokines (IL-1β, IL-8) and degradative enzymes (MMP3, ADAMTS4), and decreased the histological grade of degeneration when compared to degenerated control IVDs. All of which are indicative of the potential disease-modifying and regenerative effects of NPgel injection with or without the addition of BMPCs as a new cell source. This shows that restoration of the intradiscal pressure by means of NPgel injection can shift the catabolic and inflammatory phenotype of NP cells into a regenerative, anabolic cell type.

We have previously reported that NPgel has the capacity to differentiate incorporated BMPCs into NP-like cells without the addition of growth factors, provides protection and enables the retention of their differentiated phenotype when exposed to degenerate conditions.^47,49^ NPgel also integrates with disc tissue when injected, restores the mechanical function of degenerate tissue and has been shown to exhibit no adverse side effects.^47,63^ This highlights the potential of NPgel to become a viable treatment strategy for IVD degeneration, however, for successful translation into the clinic, further assessment prior to *in vivo* testing, is required. Therefore, we utilised our previously developed *ex vivo* LDCS^56^ to rapidly test NPgel tissue integration, disease-modifying effects, and the influence of BMPCs, within an environmentally relevant, reproducible and contained system to further determine the efficacy of our hydrogel.

Following NPgel injection into pre-degenerated IVDs and subsequent culture in the LDCS, a decrease in the expression of catabolic proteins was observed. This suggests that NPgel has the capacity to modify the progression of IVD degeneration that had been induced within the model prior to injection. Here, we observed the integration of NPgel within native tissue and an increase in matrix protein expression, which has also been reported in other systems,^64–67^ indicating the potential of these biomaterials to enable the remodelling of the degenerate IVD. We reason that the integration of NPgel, migration of native cells and increase in matrix expression indicates the regeneration of NP tissue may also be promoted by NPgel injection. Generally, other studies have cultured IVDs and biomaterials statically,^65–67^ or degeneration has been induced *via* more extreme methods than in the current study (*e.g.*, nucleotomy).^66,67^ Rosenzweig *et al.*, 2018, performed hyaluronan (HA)-hydrogel and autologous NP cell delivery into human IVDs within a loaded, physiologically relevant organ culture system, using enzymatic degradation and found that hydrogel alone accumulated proteoglycan, whereas the combination of the hydrogel and autologous NP cells produced an increase in matrix expression after 5 weeks following the onset of degeneration.^64^ These results, along with those presented here, show that within physiologically relevant organ cultures, both hydrogel systems are capable of matrix synthesis and IVD repair. However, this was only possible for the HA-hydrogel with the addition of a potentially regenerative cell source,^64^ whereas here, NPgel injection, alone or with BMPCs, also caused similar effects after a shorter culture time of 3 weeks. Indicating that NPgel itself may provide a regenerative stimulus over a shorter time frame without the need for an additional regenerative cell source. As NPgel + BMPC injection performed similarly to NPgel injection alone, this indicates the addition of this new cell source has no adverse effects on the ability of NPgel to induce regenerative changes and could potentially aid in regeneration over an extended timeframe, as the incorporation of BMPCs may still be necessary to produce the levels of matrix synthesis required over a longer period of time, and replace dead and senescent cells, which are increased during degeneration.

Here, for the first time we have shown that delivery of NPgel (±BMPCs) into a pre-induced degenerate LDCS decreases the native expression of many catabolic factors that are upregulated in the degenerate environment, suggesting that NPgel has an anti-catabolic effect. Most other studies have focused on the ability of such hydrogels to enable the differentiation of regenerative cell sources and their ability to upregulate matrix expression, without the added anti-catabolic effects.^55,64–68^ Results here, suggest that NPgel may not only have the ability to increase the production of matrix, enabling the remodelling of the IVD, but also provide protection from the catabolic environment of the degenerate IVD, by decreasing factors responsible for matrix destruction, and catabolic cytokines which can drive numerous pathogenic effects.^10,12,13,69–73^ Together, this suggests NPgel may have the ability to target IVD degeneration from both sides; to allow the production of new matrix, whilst also protecting deposited matrix from destruction.

The level of degeneration observed here in control discs is more severe than seen in our previous study after the initial 1 week of degeneration.^56^ Where, we showed that the catabolic phenotype of native cells was induced within the LDCS, but no large fissures in the tissue were present.^56^ Within the current study, control discs were cultured for a total of 4 weeks following the initial enzyme digestion under physiological loading, resulting in large fissure formation. This suggests that the initial degenerative effects observed following 1 week of physiological loading continue in untreated discs during the remaining 3 weeks of culture. Hence, we postulate, it is most likely that the results seen here, following NPgel (±BMPC) injection, are a combination of preventing further degeneration during the additional 3 weeks of culture as no large tissue fissures were present, whilst also reversing the degenerate cascade of cytokines, degradative enzymes and catabolic factors, which had been induced over the first week of culture,^56^ leading to regenerative effects.

The next steps to further elucidate the efficacy of NPgel could be to investigate any potential anti-catabolic and regenerative properties within an *in vivo* animal model of IVD degeneration. One such model has been previously developed by Hoogendoorn *et al.*, 2007 and 2008,^52,74^ which was further characterised recently,^53^ and utilises enzyme injection to cause IVD degeneration within an *in vivo* caprine system. This model also exhibits similar catabolic changes to our *ex vivo* LDCS, indicating its promising potential as a model of human degeneration and its ability to test biomaterials.^75^ Injection of a dextran, chitosan and teleostean interpenetrating network hydrogel into the *in vivo* caprine model provided an improvement in disc height and histological condition of degenerate discs after 2 weeks, yet only modest anti-catabolic effects (a decrease in TNFα expression) were observed.^76^ Coupled with the 12 weeks taken to induce degeneration in the *in vivo* model,^72^ compared to 1 week in our LDCS,^55^ it may be more pertinent in the future to utilise an *ex vivo* human model of IVD degeneration to test NPgel, rather than an *in vivo* animal model, to determine if results seen here and previously translate to modifying the phenotype of human IVD degeneration, within a fast, reproducible model. Taken together, such results may then inform the capabilities of NPgel to be taken forward to large animal testing for *in vivo* safety, prior to human trials.

## Conclusions

This study demonstrates that NPgel injected into degeneration-induced caprine IVDs exhibited the ability to enable new matrix production alongside the inhibition of the catabolic cascade associated with IVD degeneration. Interestingly, NPgel injection alone displayed the same capability as NPgel + BMPCs. This indicates that NPgel itself may be sufficient to provide anti-catabolic and tissue regenerative properties for the IVD, whilst conveying the ability to deliver, differentiate and protect a new cell source if required during longer-term IVD regeneration. Taken together, this highlights the potential restorative effects of NPgel upon IVD degeneration, and as such may be considered for future testing as a viable treatment strategy for intervertebral disc degeneration.

## Author contributions

CS, THS and CLLM contributed to the conception and design of the study and funding acquisition, JWS, CR, KSE, RJ, SP and CLLM contributed to the acquisition of laboratory data, JWS, and CLLM performed data analysis, JWS, CR, KSE, CS, THS and CLM contributed to interpretation of the data, JWS drafted the manuscript, KSE, CR, RJ, SP, CS, THS and CLM critically revised the manuscript. CLLM and THS coordinated the study. All authors read and approved the final manuscript.

## Conflicts of interest

CLLM and CS are co-inventors on a patent for the hydrogel system (NPgel) described in this paper.

## Supplementary Material

BM-011-D3BM00150D-s001

BM-011-D3BM00150D-s002
