# Supplementary material for: Injectable biomaterial induces regeneration of the intervertebral disc in a caprine loaded disc culture model
Source: Biomater Sci. 2023 May 17;11(13):4630–43. doi: 10.1039/d3bm00150d (PMC10294806; doi:10.1039/d3bm00150d)
Supplement: BM-011-D3BM00150D-s001 [file BM-011-D3BM00150D-s001.pdf]

Electronic Supplementary Material (ESI) for Biomaterials Science.
This journal is © The Royal Society of Chemistry 2023

Supplementary Figure 1: Schematic representing mechanical loading regime in LDCS: reproduced with kind permission[58].
