# Supplementary figures and images for: Injectable biomaterial induces regeneration of the intervertebral disc in a caprine loaded disc culture model
Source: Biomater Sci. 2023 May 17;11(13):4630–43. doi: 10.1039/d3bm00150d (PMC10294806; doi:10.1039/d3bm00150d)

## Simulated Physiological Loading

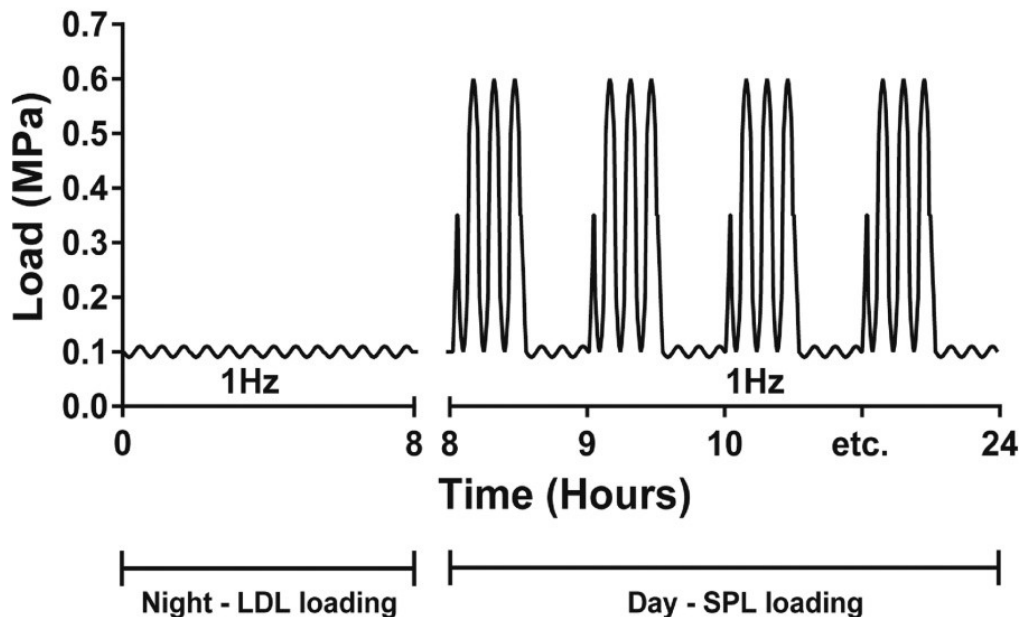

Supplement: BM-011-D3BM00150D-s002 [file BM-011-D3BM00150D-s002.pdf]
